# Sex Differences in Apolipoprotein E and Alzheimer Disease Pathology Across Ancestries

**DOI:** 10.1001/jamanetworkopen.2025.0562

**Published:** 2025-03-11

**Authors:** Xiaoyi Xu, Jiseon Kwon, Ruiqi Yan, Catherine Apio, Soomin Song, Gyujin Heo, Qijun Yang, Jigyasha Timsina, Menghan Liu, John Budde, Kaj Blennow, Henrik Zetterberg, Alberto Lleó, Agustin Ruiz, José Luis Molinuevo, Virginia Man-Yee Lee, Yuetiva Deming, Amanda J. Heslegrave, Tim J. Hohman, Pau Pastor, Elaine R. Peskind, Marilyn S. Albert, John C. Morris, Taesung Park, Carlos Cruchaga, Yun Ju Sung

**Affiliations:** 1 NeuroGenomics and Informatics Center, Washington University School of Medicine, St Louis, Missouri; 2 Division of Biostatistics, Washington University School of Medicine, St Louis, Missouri; 3 Department of Psychiatry, Washington University School of Medicine, St Louis, Missouri; 4 Interdisciplinary Program in Bioinformatics, Seoul National University, Seoul, Korea; 5 Department of Quantitative Health Sciences, Cleveland Clinic, Cleveland, Ohio; 6 Department of Psychiatry and Neurochemistry, Institute of Neuroscience and Physiology, Sahlgrenska Academy at the University of Gothenburg, Mölndal, Sweden; 7 Sant Pau Memory Unit, Institut d’Investigació Biomèdica Sant Pau (IIB SANT PAU), Barcelona, Spain; 8 Research Center and Memory Clinic, ACE Alzheimer Center Barcelona, Universitat Internacional de Catalunya, Barcelona, Spain; 9 BarcelonaBeta Brain Research Center, Pasqual Maragall Foundation, Barcelona, Spain; 10 Department of Pathology and Laboratory Medicine, Perelman School of Medicine at the University of Pennsylvania, Philadelphia; 11 Department of Medicine, School of Medicine and Public Health, University of Wisconsin, Madison; 12 Department of Neurodegenerative Disease, UCL Institute of Neurology, London, United Kingdom; 13 Vanderbilt Memory and Alzheimer’s Center, Vanderbilt University Medical Center, Nashville, Tennessee; 14 University Hospital Germans Trias i Pujol and The Germans Trias i Pujol Research Institute (IGTP), Badalona, Spain; 15 Department of Psychiatry and Behavioral Sciences, University of Washington, Seattle; 16 Department of Neurology, Johns Hopkins University School of Medicine, Baltimore, Maryland; 17 Department of Neurology, Washington University, St Louis, Missouri; 18 Knight Alzheimer’s Disease Research Center, Washington University, St Louis, Missouri; 19 Department of Statistics, Seoul National University, Seoul, Korea; 20 Hope Center for Neurological Disorders, Washington University School of Medicine, St Louis, Missouri

## Abstract

**Importance:**

Age, sex, and apolipoprotein E (*APOE*) are the strongest risk factors for late-onset Alzheimer disease (AD). The role of *APOE* in AD varies with sex and ancestry. While the association of *APOE* with AD biomarkers also varies across sex and ancestry, no study has systematically investigated both sex-specific and ancestry differences of *APOE* on cerebrospinal fluid (CSF) biomarkers together, resulting in limited insights and generalizability.

**Objective:**

To systematically investigate the association of sex and *APOE*-ε4 with 3 core CSF biomarkers across ancestries.

**Design, Setting, and Participants:**

This cohort study examined 3 CSF biomarkers (amyloid β1-42 [Aβ42], phosphorylated tau 181 [p-tau], and total tau, in participants from 20 cohorts from July 1, 1985, to March 31, 2020. These individuals were grouped into African, Asian, and European ancestries based on genetic data. Data analyses were conducted from June 1, 2023, to November 10, 2024.

**Exposure:**

Sex (male or female) and *APOE*-ε4.

**Main Outcomes and Measures:**

The associations of sex and *APOE*-ε4 with biomarker levels were assessed within each ancestry group, adjusting for age. Meta-analyses were performed to identify these associations across ancestries. Sensitivity analyses were conducted to exclude the potential influence of the *APOE*-ε2 allele.

**Results:**

This cohort study included 4592 individuals (mean [SD] age, 70.8 [10.2] years; 2425 [52.8%] female; 119 [2.6%] African, 52 [1.1%] Asian, and 4421 [96.3%] European). Higher *APOE*-ε4 dosage scores were associated with lower Aβ42 values (β [SE], −0.58 [0.02], *P* < .001), indicating more severe pathology; these associations were seen in men and women separately and jointly. The association with *APOE*-ε4 was statistically greater in men (β [SE], −0.63 [0.03]; *P* < .001) vs women (β [SE], −0.52 [0.03]; *P* < .001) of European ancestry (*P* = .01 for interaction). Women had higher levels of p-tau, indicating more severe neurofibrillary pathology. The association between *APOE*-ε4 dosage and p-tau was in the expected direction (higher *APOE*-ε4 dosage for higher p-tau values) in both sexes, but the difference between sexes was significant only in those of African ancestry (β [SE], 0.10 [0.18]; *P* = .57 for men; β [SE], 0.66 [0.17]; *P* < .001 for women; *P* = .03 for interaction). Women also had higher levels of total tau, indicating more neuronal damage. The association between *APOE*-ε4 dosage and total tau was stronger in women than in men in the African cohort (β [SE], 0.20 [0.22]; *P* = .36 for men and β [SE], 0.65 [0.22], *P* = .004 for women [*P* = .16 for interaction]) and European cohort (β [SE], 0.36 [0.03]; *P* < .001 in women and β [SE], 0.27 [0.03], *P* < .001 in men [*P* = .053 for interaction]); no significant associations were found in the Asian cohort. Sensitivity analysis excluding *APOE-*ε2 carriers yielded similar results.

**Conclusions and Relevance:**

In this cohort study, the association of the *APOE*-ε4 risk allele with tau accumulation was higher in women than in men. These findings underscore the importance of considering sex differences in *APOE*-ε4’s association with AD biomarkers and tau pathology mechanisms in AD. Although this study provides robust evidence of complex interplay between sex and *APOE*-ε4 for European ancestry, further research is needed to fully understand other ancestry differences.

## Key Points

**Question:**

How does the association of apolipoprotein E (*APOE*) with amyloid and tau pathology vary across different sex and ancestry groups?

**Findings:**

In this cohort study of 4592 individuals, *APOE*-ε4 dosage was associated with more severe amyloid β1-42 pathology with sex × *APOE*-ε4 interaction in individuals of European ancestry. The association of *APOE*-ε4 with phosphorylated tau was stronger in women than in men, specifically among individuals of African ancestry, whereas for total tau, the sex difference was significant among individuals of African and European ancestries.

**Meaning:**

These results highlight the importance of considering sex and ancestry when understanding the role of *APOE* in Alzheimer disease pathology, which may enhance insights into disease mechanisms.

## Teaser

This cohort study investigates the main and interactive associations of sex and apolipoprotein E with core cerebrospinal biomarkers in individuals of different ancestries.

## Introduction

Alzheimer disease (AD) is the leading cause of dementia worldwide and represents a significant public health concern as a major expensive and burdensome disease.^1,2,3^ The strongest risk factors for AD are advanced age, biological sex, and genetic variants.^3^ In particular, women are at a higher risk of developing AD and exhibit faster progression with more rapid cognitive decline after diagnosis compared with men.^4^ Among genetic variants, the apolipoprotein E (*APOE*) ε4 allele (*APOE*-ε4) is the largest genetic risk, explaining approximately 13% of phenotypic variance for late-onset AD.^5,6,7^ The lifetime risk of AD is more than 50% for *APOE*-ε4/ε4 and 20% to 30% for *APOE*-ε3/ε4 carriers compared with *APOE*-ε2 or *APOE*-ε3 carriers.^8,9,10^ These risks are reported to vary across ancestries.^11,12^

Among the cerebrospinal fluid (CSF) biomarkers for AD are amyloid β1-42 (Aβ42) for cortical amyloid deposition, phosphorylated tau 181 (p-tau) for neurofibrillary pathological changes, and total tau for the intensity of neurodegeneration.^13^ Although p-tau is highly specific to AD, total tau serves as a broader marker of neurodegeneration, indicating neuronal damage across both AD and non-AD conditions.^13^ These 3 CSF biomarkers have significantly increased the diagnostic accuracy of AD in the mild cognitive impairment (MCI) stage^14,15^ and sporadic AD with a combined sensitivity of greater than 95% and a specificity of greater than 85%.^13,16,17,18,19^ AD can be differentiated from other dementias by detecting lower concentrations of Aβ42 and higher concentrations of p-tau or total tau in CSF compared with age-matched control individuals.^20^

To disentangle the interplay between sex and *APOE* with AD, several studies have examined the association of *APOE* with the underlying mechanism by considering these CSF or positron emission tomography (PET) biomarkers. While *APOE*-ε4 is associated with severe amyloid and tau pathology even in cognitively unimpaired individuals,^21^ its role often differs between men and women. Several studies, including the study by Hohman et al,^22^ reported that the role of *APOE* in p-tau and total tau was more pronounced in women. Other studies, including the study by Altmann et al,^23^ also reported sex differences in the association of *APOE*-ε4 with Aβ42 in individuals with MCI. In addition, the role of *APOE* in AD pathology is known to vary across ancestries.^12,24,25,26^ The levels of p-tau and total tau in African American individuals have been reported to be lower than those in European individuals,^12,24,25,26^ with a potential ancestry × *APOE*-ε4 interaction in AD pathology.^27^ This finding emphasizes the importance of considering ancestry in AD biomarker analyses. However, to our knowledge, no study has systematically investigated both sex- and ancestry-specific differences of the association between *APOE*-ε4 and CSF biomarkers together. Therefore, our study’s objective was to investigate the main and interactive associations of sex and *APOE*-ε4 with 3 core CSF biomarkers (Aβ42, p-tau, and total tau) across individuals of 3 ancestries.

## Methods

### Study Participants

The study considered individuals recruited from July 1, 1985, to March 31, 2020, from 20 cohorts: the Alzheimer Disease Neuroimaging Initiative^28^; Australian Imaging, Biomarkers, and Lifestyle^29,30^; Biomarkers of Cognitive Decline Among Normal Individuals^31^; Blennow et al^13^; Barcelona-1^32^; Ace Alzheimer Center Barcelona, also known as Fundació ACE^33^; Homburg, Germany^34^; Hospital Sant Pau^35^; London, England^36,37,38^; Clinic de Barcelona^35^; the Knight Alzheimer Disease Research Center^39,40^; the Mayo Clinic Study of Aging^41^; National Alzheimer Coordinating Center^42^; Skåne University Hospital^43^; Wisconsin Alzheimer Disease Research Center^44^; University of Pennsylvania^42,43^; University of Washington^43^; the Vanderbilt Memory and Aging Project^45^; Mattsson et al,^20^ and the Wisconsin Registry for Alzheimer’s Prevention.^46^ The cohort-specific description and characteristics are presented in the eMethods and eTable 1 in Supplement 1. Written informed consent was obtained from all study participants. For those with substantial cognitive impairment, consent was obtained from a caregiver or legal guardian. The appropriate institutional review boards evaluated and approved the study protocols for all cohorts. The present study was approved by the Washington University School of Medicine institutional review board and followed the Strengthening the Reporting of Observational Studies in Epidemiology (STROBE) reporting guideline.

### Ancestry Classification

From 20 cohorts, 4630 individuals with and without AD were considered. After excluding individuals with missing data on biomarkers, sex, age, or *APOE* genotype, 4592 remained (more details are presented in eFigure 1 in Supplement 1). To classify these individuals into ancestry, we performed principal component analysis by combining genomic data from these 4592 individuals and HapMap data. On the basis of the first 2 principal component analyses, individuals clustered near Europe were classified as having European ancestry (n = 4421) (eFigure 2 in Supplement 1). The remaining individuals were classified as having African (n = 119) or Asian (n = 52) ancestry.

### CSF Biomarkers

This study examined 3 core CSF AD biomarkers: Aβ42, p-tau, and total tau. The CSF biomarker protein levels from the 20 cohorts were obtained using different platforms in each cohort. For details on specific laboratory procedures, see the original studies.^20,28,29,30,31,32,33,35,39,40,41,42,43,45,46^ To ensure comparability among the different cohorts and to reduce batch effects, we harmonized the CSF biomarker values across cohorts, as described previously.^47^ Briefly, the CSF biomarker levels were log_10_ transformed and *z* score normalized in each cohort. Any biomarker values outside 3 SDs from the mean were considered outliers and excluded from the analysis. The distribution of these CSF values before and after harmonization is presented in eFigure 3 in Supplement 1.

### APOE Genotyping

The *APOE* haplotypes (ε2, ε3, and ε4) on chromosome 19 were determined using rs7412 and rs429358 genotypes. To capture the effect of the number of ε4 alleles, the *APOE*-ε4 dosage was coded as a continuous variable: 0 for individuals with no copy (ε2/ε2, ε2/ε3, and ε3/ε3), 1 for individuals with 1 copy (ε3/ε4 and ε2/ε4), and 2 for individuals with 2 copies (ε4/ε4).

### Statistical Analysis

We combined harmonized data from all cohorts and split them into each ancestry. For each ancestry, we performed the following 4 regression analyses:

*Y* = α + β_1_ × *APOE*-ε4 + β_2_ × Sex + β_3_ × Age (in both sexes);

*Y* = α + β_1_ × *APOE-*ε4 + β_2_ × Age (in men only);

*Y* = α + β_1_ × *APOE*-ε4 + β_2_ × Age (in women only); and

*Y* = α + β_1_ × *APOE*-ε4 + β_2_ × Sex + β_3_ × Age + β_4_ × *APOE*-ε4 × Sex (in both sexes).

Overall sex difference (β_1_) and *APOE-*ε4 association (β_2_) were obtained in model 1, considering both sexes. The sex-specific *APOE-*ε4 association (β_2_) was obtained via models 2 and 3 (male specific in model 2; female specific in model 3). Finally, the interaction between *APOE*-ε4 allele and sex (β_4_) was obtained via model 4 to test the difference between the sex-specific *APOE*-ε4 associations. *Y* was the normalized level of CSF Aβ42, p-tau, and total tau. Sex was coded as 0 for men and 1 for women. *APOE*-ε4 was the dosage (count) of the ε4 allele in *APOE*-ε4 genotype (0, 1, or 2). In addition, age at the time of the CSF draw was included as a covariate to adjust for the association of age with these CSF biomarkers.^48,49^

To examine whether the resulting *APOE*-ε4 association was confounded by the known protective ε2 effect, we conducted 2 sensitivity analyses. The first sensitivity analysis considered only ε3/ε3 carriers for 0 (excluding ε2/ε2 and ε2/ε3). The second sensitivity analysis completely excluded any ε2 carriers, thereby considering 0 for ε3/ε3, 1 for ε3/ε4, and 2 for ε4/ε4. We then performed a meta-analysis to estimate their overall associations across the population using the inverse variance weighted method. Heterogeneity was assessed with the Cochran *Q* test^50^ in all meta-analyses, and when *P* < .05, both the fixed-effect and random-effect models were reported.

All analyses were performed using R, version 4.3.1 (R Foundation for Statistical Computing). Meta-analysis was conducted using the meta R package.^51^ Forest plots for visualizing meta-analysis results were generated using the metafor R package.^52^ A 2-sided *P* < .05 was considered statistically significant. Data analyses were conducted from June 1, 2023, to November 10, 2024.

## Results

### Participant Characteristics

The characteristics of the 4592 participants examined in this study are summarized in Table 1. The mean (SD) age was 70.8 (10.2) years, reflecting an older population relevant to AD. Approximately half of the European and Asian participants (2171 [49.1%] and 27 [51.9%], respectively) were clinically diagnosed with AD, whereas 31.9% of African participants (n = 38) were diagnosed with AD. A total of 2425 participants (52.8%) were female, and 2167 (47.2%) were male. European and African ancestry had a slightly higher representation of women (74 [62.2%] in the African cohort, 22 [42.3%] in the Asian cohort, and 2329 [52.7%] in the European cohort). *APOE*-ε4 carriers were more prevalent in the European cohort compared with other ancestries (34.5% in African, 25.0% in Asian, and 41.0% in European), reflecting population differences in *APOE* allele frequencies. Although the median values of CSF biomarkers varied among the 3 ancestries, these values were not statistically different. Cohort-specific characteristics are presented in eTable 1 in Supplement 1 for demographic characteristics, eTable 2 in Supplement 1 for *APOE* genotypes, and eTable 3 in Supplement 1 for biomarker distributions.

**Table 1.** Participant Characteristics

Characteristic	No. (%) of participants^a^		
	European (n = 4421)	African (n = 119)	Asian (n = 52)
Age, mean (SD), y	71.0 (10.3)	69.0 (7.6)	69.0 (13.3)
Sex			
Male	2092 (47.3)	45 (37.8)	30 (57.7)
Female	2329 (52.7)	74 (62.2)	22 (42.3)
Clinical diagnosis			
AD	2171 (49.1)	38 (31.9)	27 (51.9)
Control	2250 (50.9)	81 (68.1)	25 (48.1)
*APOE* genotype			
22	6 (0.1)	4 (3.4)	NA (0.0)
23	358 (8.1)	9 (7.6)	6 (11.5)
24	85 (1.9)	2 (1.7)	NA (0.0)
33	2243 (50.7)	65 (54.6)	33 (63.5)
34	1396 (31.6)	32 (26.9)	8 (15.4)
44	333 (7.5)	7 (5.9)	5 (9.6)
*APOE*-ε4 carrier			
0	2607 (59.0)	78 (65.5)	39 (75.0)
1	1481 (33.5)	34 (28.6)	8 (15.4)
2	333 (7.5)	7 (5.9)	5 (9.6)
Aβ42, median (IQR), pg/mL	340 (183-639)	276 (192-726)	215 (157-266)
Phosphorylated tau, median (IQR), pg/mL	40 (25-65)	30 (23-45)	35 (23-54)
Total tau, median (IQR), pg/mL	179 (77-412)	116 (50-232)	63 (40-120)

Abbreviations: Aβ42, amyloid β1-42; AD, Alzheimer disease; APOE, apolipoprotein E.

^a^ Unless otherwise indicated. Mean (SD) for Aβ42, phosphorylated tau, and total tau biomarkers, as well as the median (IQR) for log_10_-transformed Aβ42, phosphorylated tau, and total tau values, were calculated based on samples stratified by ancestry.

### Sex Differences in CSF Biomarker Levels

We first examined sex differences in CSF biomarkers before examining the *APOE* association in model 1. For CSF Aβ42 levels, no significant sex association was observed, indicating similar levels in men and women across all 3 ancestries (β [SE], 0.27 [0.19]; *P* = .14 for African; β [SE], −0.28 [0.32]; *P* = .004 for Asian; and β [SE], −0.00 [0.03]; *P* = .92 for European ancestry), with no evidence of heterogeneity (eTables 4 and 5 in Supplement 1).

For CSF p-tau, women had significantly higher levels than men in both the European and African ancestry cohorts (β [SE], 0.08 [0.03]; *P* = .007 in the European cohort; β [SE], 0.45 [0.17]; *P* = .001 in the African cohort) (eTable 4 in Supplement 1). A similar finding was observed for Asian ancestry (β [SE], 0.60 [0.32]; *P* = .431), but it was not significant due to the small sample size. This was consistent across all 3 ancestries, although the ancestry-specific effect size showed evidence of heterogeneity (European individuals having a smaller effect size compared with African and Asian individuals; *P* = .048 for heterogeneity).

For CSF total tau, women consistently had higher levels than men in the European and African cohorts, but the effect size was not significant in the Asian cohort (β [SE], 0.55 [0.21]; *P* = .009 in the African cohort; β [SE], 0.60 [0.35]; *P* = .360 in the Asian cohort; and β [SE], 0.12 [0.03]; *P* < .001 in the European cohort) (eTable 4 in Supplement 1). In the meta-analysis, the overall sex difference across 3 ancestries was significant (β [SE], 0.12 [0.03]; *P* < .001), with no evidence of heterogeneity (*P* = .06 for heterogeneity).

### Association of *APOE*-ε4 With CSF Biomarker Levels

For all 3 biomarkers, we observed a significant association with *APOE*-ε4 (Table 2). Relative to the European cohort (β [SE], −0.57 [0.02]; *P* < .001), this *APOE*-ε4 association was somewhat stronger in African and Asian ancestries (β [SE], −0.75 [0.14]; *P* < .001 in the African cohort; β [SD], −0.69 [0.23]; *P* = .004 in the Asian cohort) (eFigure 4 in Supplement 1), although statistical significance was reduced due to relatively fewer samples. The overall *APOE*-ε4 association remained significant in the meta-analysis (β [SE], −0.58 [0.02]; *P* < .001) with no evidence of heterogeneity.

**Table 2.** *APOE*-ε4 Effect Within and Across Ancestry^a^

Trait	European		African		Asian		Meta-analysis across ancestries	
	β (SE)	*P* value	β (SE)	*P* value	β (SE)	*P* value	β (SE)	*P* value
Aβ42								
Both sexes	−0.57 (0.02)	<.001	−0.75 (0.14)	<.001	−0.69 (0.23)	.004	−0.58 (0.02)	<.001
Male	−0.63 (0.03)	<.001	−0.81 (0.23)	.001	0.34 (0.61)	.58	−0.63 (0.03)	<.001
Female	−0.52 (0.03)	<.001	−0.66 (0.17)	<.001	−0.86 (0.22)	<.001	−0.53 (0.03)	<.001
Sex × *APOE*-ε4 interaction	0.11 (0.04)	.01	0.17 (0.28)	.55	−1.24 (0.62)	.049	0.11 (0.04)	.01
Phosphorylated tau								
Both sexes	0.35 (0.02)	<.001	0.44 (0.13)	<.001	0.18 (0.23)	.43	0.35 (0.02)	<.001
Male	0.33 (0.03)	<.001	0.10 (0.18)	.57	−0.69 (0.49)	.17	0.32 (0.03)	<.001
Female	0.37 (0.03)	<.001	0.66 (0.17)	<.001	0.34 (0.30)	.27	0.38 (0.03)	<.001
Sex × *APOE*-ε4 interaction	0.03 (0.05)	.48	0.55 (0.26)	.03	1.04 (0.63)	.11	0.34 (0.26)	.19
Tau								
Both sexes	0.32 (0.02)	<.001	0.46 (0.16)	.004	0.25 (0.27)	.36	0.32 (0.02)	<.001
Male	0.27 (0.03)	<.001	0.20 (0.22)	.36	−0.90 (0.56)	.12	0.27 (0.03)	<.001
Female	0.36 (0.03)	<.001	0.65 (0.22)	.004	0.48 (0.34)	.18	0.37 (0.03)	<.001
Sex × *APOE*-ε4 interaction	0.09 (0.05)	.053	0.46 (0.32)	.16	1.32 (0.73)	.08	0.10 (0.05)	.03

Abbreviations: Aβ42, amyloid β1-42; *APOE*, apolipoprotein E.

^a^ *APOE*-ε4 evaluates the main effect of the *APOE*-ε4 allele on cerebrospinal fluid biomarkers from model 1. Values for male participants focus on the association of *APOE*-ε4 in male participants from model 2. Values for female participants focus on the association of *APOE*-ε4 in female participants from model 3. Interaction values focus on the interaction between sex and *APOE*-ε4 on cerebrospinal fluid biomarkers from model 4.

*APOE*-ε4 carriers had higher tau levels, with significant associations seen in the European and African cohorts for p-tau (β [SE], 0.35 [0.02]; *P* < .001 in the European cohort; β [SE], 0.44 [0.13]; *P* < .001 in the African cohort) as well as for total tau (β [SE], 0.32 [0.02]; *P* < .001 in the European cohort and β [SE], 0.46 [0.16]; *P* = .004 for the African cohort). In the Asian cohort, there was no significant association for p-tau or total tau due to the small sample size. A meta-analysis confirmed an overall significant association of APOE-ε4 with both p-tau and total tau values (β [SE], 0.35 [0.02]; *P* < .001 for p-tau and 0.32 [0.02]; *P* < .001 for total tau).

### Sex-Specific *APOE*-ε4 Association

To examine the *APOE*-ε4 association in more detail, we analyzed men and women separately using models 2 and 3 and then confirmed sex differences through the significance of the interaction term in model 4. The association of *APOE*-ε4 with Aβ42 was more pronounced in European men (β [SE], −0.63 [0.03]; *P* < .001) compared with women (β [SE], −0.52 [0.03]; *P* < .001) (Figure, A and eFigure 5 in Supplement 1), with a statistically significant difference (*P* = .01 for interaction) (Table 2). A similar trend was observed in African ancestry. In Asian ancestry, the *APOE*-ε4 association was only observed in women, whereas the result for men was not in the expected direction due to a very small sample of *APOE*-ε4 carriers (n = 3). A meta-analysis confirmed a significant interaction between sex and *APOE*-ε4 on Aβ42 (β [SE], 0.11 [0.04]; *P* = .01 for interaction) (eFigure 6 in Supplement 1).

**Figure.**
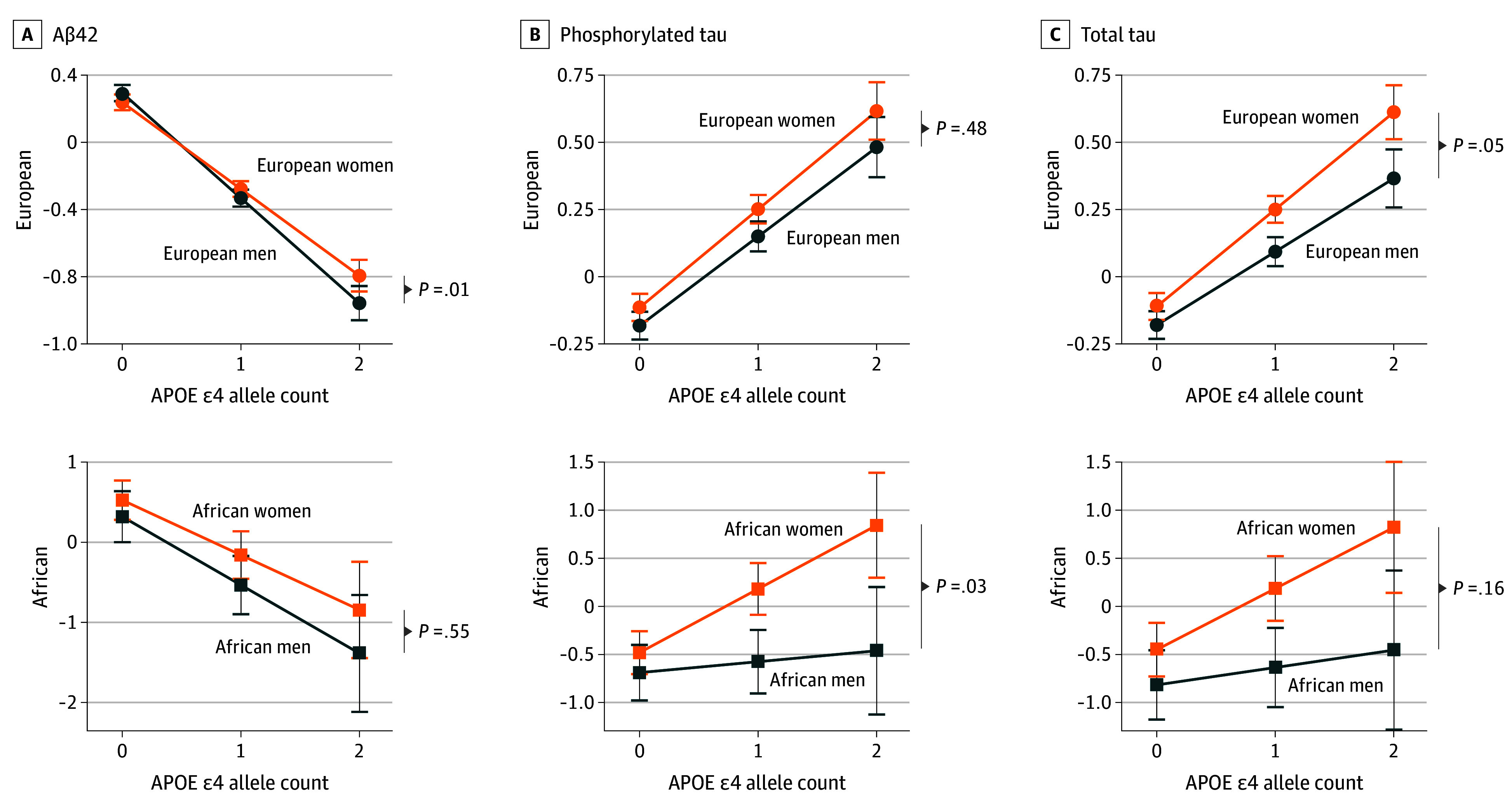
Sex-Specific *APOE*-ε4 Associations With Cerebrospinal Fluid Biomarkers in Individuals of European and African Ancestry Sex-stratified results are shown for each *APOE*-ε4 dosage with 95% CIs. Statistical models were adjusted for age. *P* value is from sex × *APOE*-ε4 interaction within each ancestry. Aβ42 indicates amyloid β1-42; *APOE*, apolipoprotein E.

For p-tau, the association with *APOE*-ε4 differed between men and women only in individuals of African ancestry (Figure, B). In the European ancestry cohort, the association with *APOE*-ε4 was somewhat higher in women (β [SE], 0.37 [0.03] in women; β [SE], 0.33 [0.03] in men) but not significantly different (*P* = .48 for interaction). In the African ancestry cohort, the *APOE*-ε4 association was observed in women (β [SE], 0.66 [0.17]; *P* < .001) but not men (β [SE], 0.10 [0.18]; *P* = .57), with a significant difference (*P* = .03 for interaction). In the Asian ancestry cohort, the *APOE*-ε4 association was not significant in either sex (eFigure 7 in Supplement 1). Due to this heterogeneity in differences across ancestries (*P* = .04 for heterogeneity), no overall interaction was observed.

For total tau, the *APOE*-ε4 association was stronger in women for all 3 ancestries. In the European ancestry cohort, this difference (β [SE], 0.36 [0.03]; *P* < .001 in women; β [SE], 0.27 [0.03]; *P* < .001 in men) was not significant (*P* = .053 for interaction). In the African ancestry cohort, the *APOE*-ε4 association was observed in women (β [SE], 0.65 [0.22]; *P* = .004) but not men (β [SE], 0.20 [0.22]; *P* = .36), although this difference was not statistically significant (*P* = .16 for interaction). In the Asian ancestry cohort, no significant associations were found. Although the magnitudes of these associations were somewhat different, there was no evidence of heterogeneity across ancestries (*P* = .13 for heterogeneity). A meta-analysis confirmed a significant overall interaction between sex and *APOE*-ε4 on total tau (β [SE], 0.10 [0.05]; *P* = .03 for interaction).

### Sensitivity Analysis

Our analysis included *APOE*-ε2 carriers in the reference group (*APOE*-ε4 dosage = 0) as well as *APOE*-ε4 dosage 1 group. To examine whether our results were influenced by inclusion of any *APOE*-ε2 carriers, we conducted 2 sets of sensitivity analyses. The first sensitivity analysis that included only ε3/ε3 carriers as the reference group (coded as 0; excluding ε2/ε2 and ε2/ε3) was highly similar to the primary analysis (eTable 6 in Supplement 1). The second sensitivity analysis that completely excluded any ε2 carriers (0 for ε3/ε3, 1 for ε3/ε4, and 2 for ε4/ε4) was also similar, with a slight difference in their *P* values due to reduced sample size (eTable 7 in Supplement 1). In particular, the sex × *APOE*-ε4 interaction on Aβ in the European cohort was identical across the 3 sets (β [SE], 0.11 [0.04]) (Table 2; eTables 6 and 7 in Supplement 1). We observed consistent interactions for total tau in the European cohort (β [SE], 0.09 [0.05]) and a similar interaction for p-tau in the African cohort (β [SE], 0.55 [0.26] in the primary analysis and first sensitivity analysis and 0.54 [0.28] in the second sensitivity analysis). These analyses demonstrated that the inclusion of *APOE*-ε2 did not alter the observed sex differences.

## Discussion

This study, to our knowledge, is the first to evaluate the main and interactive associations of sex and *APOE*-ε4 dosage with 3 CSF biomarkers across African, Asian, and European ancestries. This study harmonized CSF biomarkers of 4592 participants recruited from 20 cohorts and performed ancestry-specific analyses and meta-analysis, providing robust evidence of complex interplay among sex, ancestry, and *APOE*-ε4 in these AD biomarkers. First, for CSF Aβ42, a higher *APOE*-ε4 dosage score was associated with lower values, indicating more severe Aβ pathology, in men and women separately and jointly. The male and female *APOE*-ε4 associations were statistically different in those of European ancestry. Second, for CSF p-tau, women had higher levels, indicating more severe neurofibrillary pathology. Although the association between *APOE*-ε4 dosage and p-tau was in the expected direction (higher *APOE*-ε4 dosage for higher p-tau values) in both sexes, the association was statistically different only in those of African ancestry. Third, for CSF total tau, women had higher levels, indicating more neuronal damage. Moreover, the association between *APOE*-ε4 dosage and total tau was stronger in women than men in both the African and European cohorts.

Several studies have investigated the association of sex and *APOE*-ε4 with Aβ and tau pathology. Altmann et al^23^ reported the sex × *APOE*-ε4 interactions for Aβ in healthy individuals and interaction for p-tau and total tau in individuals with MCI, suggesting that interaction between sex and *APOE* depends on cognition status. Hohman et al^22^ reported significant sex × *APOE*-ε4 interactions for p-tau and total tau, among which interaction remained significant for total tau in Aβ-positive individuals. Although our finding for sex × *APOE*-ε4 interactions on Aβ is different from that of Hohman et al,^22^ it is in line with the finding of Altmann et al.^23^ In addition, Saunders et al^53^ observed high negative correlation between Aβ and p-tau, with more pronounced sex differences, in *APOE*-ε4 carriers, whereas correlation in noncarriers was opposite. In contrast, multiple studies presented sex differences in the association of *APOE*-ε4 dosage with tau accumulation, regardless of amyloid levels, in individuals with MCI and AD,^54^ reporting higher tau load only in women via positron emission tomography^55^ or via CSF biomarkers.^56^ Our findings of higher p-tau and total tau levels in female *APOE*-ε4 carriers are consistent with these studies. These studies, together with ours, demonstrate a complex presentation of sex × *APOE*-ε4 interactions for AD pathology and the resulting cognition status.

Previous studies highlighted significant variation in the effect of *APOE*-ε4 on AD risk across different ethnic and racial groups, underscoring the importance of ancestry in AD studies.^11,12,57^ A recent study reported these ethnic variations for AD risk between European and African ancestry in large scale.^12^ The association of *APOE* genotype with AD neuropathology also differs by ancestry, with African individuals often exhibiting lower amyloid burden and attenuated *APOE*-ε4 risk compared with individuals of European ancestry.^58^ These ancestry differences in the association of *APOE*-ε4 with amyloid pathology appear to be confounded by age.^59^ Racial disparities in *APOE*-ε4 were also reported for tau pathology. African American participants were reported to exhibit lower CSF p-tau and total tau levels compared with White individuals,^27^ even when they had similar cognitive scores.^26^ Although one study reported these ancestry differences via genetically derived ancestry information,^60^ another study noted these ancestry differences only with self-reported race (and not in genetic ancestry).^61^ Because self-reported African individuals often exhibit a continuum of African ancestry with a large admixture with European ancestry,^60^ inconsistencies between these 2 studies may be due to their sample selection differences. In our study, relatively lower p-tau and total tau levels were noticeable in men of African ancestry, which may have resulted in sex differences in their *APOE*-ε4 effects on p-tau levels.

Recent findings have expanded the sex differences in *APOE*-ε2 and their role in AD risk and cognitive decline. The protective *APOE*-ε2 effect was reported for amyloid and regional tau burden.^21^ One study reported the association of *APOE*-ε2 with a slower cognitive decline more in men, suggesting sex-specific protective effects.^62^ Another study reported the protective effect of *APOE*-ε2 on late-life cognition in women but not men.^63^ This *APOE*-ε2 effect on AD is reported to vary across populations because it is shown to be most protective in European populations followed by African populations, with no association in Asian and Hispanic populations.^12^ Among cognitively unimpaired individuals, the protective effect of *APOE*-ε2 on baseline executive function was reported to be female specific in European individuals but male specific in African individuals.^64^ Although our study focused on *APOE*-ε4, we performed 2 sets of sensitivity analyses that examined the potential influence of any *APOE*-ε2 carriers. Both sets of analysis had highly consistent outcomes with the primary analysis, indicating that the inclusion of *APOE*-ε2 carriers does not substantially alter observed sex differences in *APOE*-ε4 association and the interplay with sex and ancestry.

There are several potential mechanisms underlying a direct or regulatory association of *APOE*-ε4 expression with CSF biomarkers and AD risk. The most prevailing explanation is through regulation of sex hormones or through sex chromosome. In women, changes in sex hormones in menopause may contribute to AD pathology and cognitive impairment.^8^ For example, estrogen loss in menopausal women may be one explanation for a more pronounced sex difference in *APOE*-ε4 associations with AD between the ages of 55 and 70 years.^65^ Estradiol has been shown to increase CSF tau accumulation while simultaneously reducing neuronal Aβ generation and preventing tau phosphorylation, indicating a multifaceted role in tau and amyloid regulation.^66,67^ *APOE* expression may also regulate the estrogen receptor α polymorphisms, which are associated with faster cognitive decline in female patients with AD.^68^ Beyond estradiol, testosterone also plays a key role in the greater CSF p-tau level in female *APOE*-ε4 carriers.^69^ In addition, sex-specific effects of *APOE*-ε4 in AD may be influenced by X chromosome mechanisms, in which differences in gene expression related to sex hormones could affect how AD develops in men and women.^70^

### Limitations

This study has several limitations. First, African and Asian ancestries had smaller sample sizes due to underrepresentation of CSF samples in non-European populations. Although the magnitude of sex × *APOE*-ε4 interaction on all 3 biomarkers was more pronounced in African and Asian ancestries (compared with those of European ancestry), statistical significance was often not reached due to the limited sample sizes. Although our results indicate lower p-tau and total tau levels in African individuals compared with European individuals, often this led to a lack of sex differences. In addition, the results in Asian men were somewhat unexpected, which appears to be affected by a limited sample size in *APOE*-ε4 carriers. Given the increasing global AD risk, the generalizability of CSF biomarkers in these populations is critical for disease prevention, warranting a follow-up study. Second, interplay between sex and *APOE*-ε4 on the cascade of amyloid pathology and tau pathology is known to be complex, often exhibiting more pronounced sex differences in certain age ranges. To disentangle our findings (in particular, the presence of sex × *APOE*-ε4 interaction on Aβ and absence on p-tau), more detailed follow-up study may be needed. Third, although this study performed the sensitivity analysis for the inclusion of *APOE*-ε2, the main scope of this study was to examine the risk effect of *APOE*-ε4. Additional research is needed to investigate the protective effects of *APOE*-ε2 on these CSF biomarkers.

## Conclusions

In this study, we report sex differences observed in the association of the *APOE*-ε4 allele with amyloid and tau pathology in individuals of African, Asian, and European ancestry based on harmonized CSF biomarkers. This study highlights the importance of accounting for sex in examining *APOE*-ε4’s influence for understanding underlying amyloid and tau pathology in AD. Although we provided robust evidence of complex interplay between sex and *APOE*-ε4 for European ancestry, further research is needed to fully understand some ancestry differences.

## Notes

Supplement 1. **eTable 1.** Demographic Distribution by Study Cohort **eTable 2.** Distribution of APOE Genotypes Across Cohorts **eTable 3.** Distribution of CSF Biomarkers (Aβ42, pTau, and Tau) by Study Cohort **eTable 4. **Sex Effect in Each Ancestry and Across Ancestry (via Meta-Analysis) **eTable 5. **Heterogeneity Test **eTable 6.** Sensitivity Analysis With ε3/ε3 as Reference **eTable 7. **Sensitivity Analysis Excluding APOE-ε2/ε4 Heterozygotes **eFigure 1.** Flowchart **eFigure 2. **Genetically Derived Ancestry Information **eFigure 3.** Histogram of CSF Biomarkers Before and After Harmonization **eFigure 4. **Sex and APOE-ε4 Effect Across Both Sexes **eFigure 5.** Sex-Specific APOE-ε4 Effects **eFigure 6.** Interaction Effect Between Sex and APOE-ε4 **eFigure 7.** Sex-Stratified APOE-ε4 Effect in Asian Ancestry **eMethods.** Cohort Description

Supplement 2.
Data Sharing Statement
