# Supplementary material for: Sex Differences in Apolipoprotein E and Alzheimer Disease Pathology Across Ancestries
Source: JAMA Netw Open. 2025 Mar 11;8(3):e250562. doi: 10.1001/jamanetworkopen.2025.0562 (PMC11897841; doi:10.1001/jamanetworkopen.2025.0562)
Supplement: Supplement 1. — eTable 1. Demographic Distribution by Study Cohort eTable 2. Distribution of APOE Genotypes Across Cohorts eTable 3. Distribution of CSF Biomarkers (Aβ42, pTau, and Tau) by Study Cohort eTable 4. Sex Effect in Each Ancestry and Across Ancestry (via Meta-Analysis) eTable 5. Heterogeneity Test eTable 6. Sensitivity Analysis With ε3/ε3 as Reference eTable 7. Sensitivity Analysis Excluding APOE-ε2/ε4 Heterozygotes eFigure 1. Flowchart eFigure 2. Genetically Derived Ancestry Information eFigure 3. Histogram of CSF Biomarkers Before and After Harmonization eFigure 4. Sex and APOE-ε4 Effect Across Both Sexes eFigure 5. Sex-Specific APOE-ε4 Effects eFigure 6. Interaction Effect Between Sex and APOE-ε4 eFigure 7. Sex-Stratified APOE-ε4 Effect in Asian Ancestry eMethods. Cohort Description [file jamanetwopen-e250562-s001.pdf]

Supplemental Online Content

Xu X, Kwon J, Yan R, et al. Sex differences in apolipoprotein E and Alzheimer disease pathology across ancestries. *JAMA Netw Open*. Published online March 11, 2025.
doi:10.1001/jamanetworkopen.2025.0562

eTable 1. Demographic Distribution by Study Cohort
eTable 2. Distribution of APOE Genotypes Across Cohorts
eTable 3. Distribution of CSF Biomarkers (Aβ42, pTau, and Tau) by Study Cohort
eTable 4. Sex Effect in Each Ancestry and Across Ancestry (via Meta-Analysis)
eTable 5. Heterogeneity Test
eTable 6. Sensitivity Analysis With ε3/ε3 as Reference
eTable 7. Sensitivity Analysis Excluding APOE-ε2/ε4 Heterozygotes
eFigure 1. Flowchart
eFigure 2. Genetically Derived Ancestry Information
eFigure 3. Histogram of CSF Biomarkers Before and After Harmonization
eFigure 4. Sex and APOE-ε4 Effect Across Both Sexes
eFigure 5. Sex-Specific APOE-ε4 Effects
eFigure 6. Interaction Effect Between Sex and APOE-ε4
eFigure 7. Sex-Stratified APOE-ε4 Effect in Asian Ancestry
eMethods. Cohort Description

This supplemental material has been provided by the authors to give readers additional information about their work.

eTable 1. Demographic distribution by study cohort

Study	N	Sex		Age, mean (SD)	Clinical diagnosis	
		Male (%)	Female (%)		AD (%)	Control (%)
**ADNI**	1197	668 (55.8)	529 (44.2)	73 (7.3)	841 (70.3)	356 (29.7)
**AIBL**	164	80 (48.8)	84 (51.2)	73 (5.7)	23 (14)	141 (86)
**Barcelona1**	59	26 (44.1)	33 (55.9)	70 (8.5)	58 (98.3)	1 (1.7)
**BIOCARD**	20	7 (35)	13 (65)	58 (5.8)	0 (0)	20 (100)
**Blennow**	56	15 (26.8)	41 (73.2)	83 (10.2)	33 (58.9)	23 (41.1)
**FACE**	349	146 (41.8)	203 (58.2)	72 (8.1)	226 (64.8)	123 (35.2)
**Homburg**	97	53 (54.6)	44 (45.4)	67 (9.2)	97 (100)	0 (0)
**Knight ADRC**	589	275 (46.7)	314 (53.3)	71 (7.4)	219 (37.2)	370 (62.8)
**Lleo**	115	43 (37.4)	72 (62.6)	64 (9.8)	47 (40.9)	68 (59.1)
**London**	81	42 (51.9)	39 (48.1)	69 (9.4)	60 (74.1)	21 (25.9)
**MAYO**	346	202 (58.4)	144 (41.6)	78 (5.4)	0 (0)	346 (100)
**Molineuvo**	182	67 (36.8)	115 (63.2)	65 (8.3)	132 (72.5)	50 (27.5)
**NACC**	286	125 (43.7)	161 (56.3)	72 (8.3)	165 (57.7)	121 (42.3)
**Sweden**	283	104 (36.7)	179 (63.3)	76 (13.4)	283 (100)	0 (0)
**Upenn**	12	3 (25)	9 (75)	69 (8.9)	0 (0)	12 (100)
**UW**	218	98 (45)	120 (55)	57 (17.4)	0 (0)	218 (100)
**VMAP**	74	52 (70.3)	22 (29.7)	72 (6.5)	0 (0)	74 (100)
**WiscADRC**	199	66 (33.2)	133 (66.8)	62 (9)	27 (13.6)	172 (86.4)
**WRAP**	222	75 (33.8)	147 (66.2)	62 (6.8)	0 (0)	222 (100)
**Zetter**	43	20 (46.5)	23 (53.5)	74 (4.3)	25 (58.1)	18 (41.9)
**Total**	**4592**	**2167 (47.2)**	**2425 (52.8)**	**71 (10.2)**	**2236 (48.7)**	**2356 (51.3)**

Abbreviations: SD, standard deviation. Means and standard deviations (SD) for age, was calculated based on samples by cohorts.

© 2025 Xu X et al. *JAMA Network Open*.

eTable 2. Distribution of APOE genotypes across cohorts

Study	APOE						APOE ε4 count		
	ε2/ε2 (%)	ε2/ε3 (%)	ε2/ε4 (%)	ε3/ε3 (%)	ε3/ε4 (%)	ε4/ε4 (%)	0 ε4 Alleles (%)	1 ε4 Alleles (%)	2 ε4 Alleles (%)
**ADNI**	2 (0.2)	96 (8)	13 (1.1)	637 (53.2)	348 (29.1)	101 (8.4)	735 (61.4)	361 (30.2)	101 (8.4)
**AIBL**	0 (0)	21 (12.8)	4 (2.4)	96 (58.5)	41 (25)	2 (1.2)	117 (71.3)	45 (27.4)	2 (1.2)
**Barcelona1**	0 (0)	2 (3.4)	2 (3.4)	19 (32.2)	35 (59.3)	1 (1.7)	21 (35.6)	37 (62.7)	1 (1.7)
**BIOCARD**	0 (0)	2 (10)	0 (0)	11 (55)	6 (30)	1 (5)	13 (65)	6 (30)	1 (5)
**Blennow**	0 (0)	6 (10.7)	2 (3.6)	33 (58.9)	15 (26.8)	0 (0)	39 (69.6)	17 (30.4)	0 (0)
**FACE**	0 (0)	27 (7.7)	6 (1.7)	186 (53.3)	108 (30.9)	22 (6.3)	213 (61)	114 (32.7)	22 (6.3)
**Homburg**	0 (0)	11 (11.3)	2 (2.1)	34 (35.1)	37 (38.1)	13 (13.4)	45 (46.4)	39 (40.2)	13 (13.4)
**Knight ADRC**	4 (0.7)	63 (10.7)	13 (2.2)	292 (49.6)	187 (31.7)	30 (5.1)	359 (61)	200 (34)	30 (5.1)
**Lleo**	0 (0)	11 (9.6)	1 (0.9)	59 (51.3)	37 (32.2)	7 (6.1)	70 (60.9)	38 (33)	7 (6.1)
**London**	0 (0)	3 (3.7)	1 (1.2)	39 (48.1)	25 (30.9)	13 (16)	42 (51.9)	26 (32.1)	13 (16)
**MAYO**	1 (0.3)	39 (11.3)	11 (3.2)	221 (63.9)	68 (19.7)	6 (1.7)	261 (75.4)	79 (22.8)	6 (1.7)
**Molineuvo**	0 (0)	5 (2.7)	2 (1.1)	104 (57.1)	59 (32.4)	12 (6.6)	109 (59.9)	61 (33.5)	12 (6.6)
**NACC**	1 (0.3)	14 (4.9)	3 (1)	127 (44.4)	112 (39.2)	29 (10.1)	142 (49.7)	115 (40.2)	29 (10.1)
**Sweden**	0 (0)	3 (1.1)	8 (2.8)	68 (24)	141 (49.8)	63 (22.3)	71 (25.1)	149 (52.7)	63 (22.3)
**Upenn**	0 (0)	1 (8.3)	0 (0)	8 (66.7)	3 (25)	0 (0)	9 (75)	3 (25)	0 (0)
**UW**	1 (0.5)	19 (8.7)	5 (2.3)	131 (60.1)	55 (25.2)	7 (3.2)	151 (69.3)	60 (27.5)	7 (3.2)
**VMAP**	1 (1.4)	6 (8.1)	1 (1.4)	44 (59.5)	19 (25.7)	3 (4.1)	51 (68.9)	20 (27)	3 (4.1)
**WiscADRC**	0 (0)	19 (9.5)	7 (3.5)	92 (46.2)	61 (30.7)	20 (10.1)	111 (55.8)	68 (34.2)	20 (10.1)
**WRAP**	0 (0)	22 (9.9)	5 (2.3)	122 (55)	66 (29.7)	7 (3.2)	144 (64.9)	71 (32)	7 (3.2)
**Zetter**	0 (0)	3 (7)	1 (2.3)	18 (41.9)	13 (30.2)	8 (18.6)	21 (48.8)	14 (32.6)	8 (18.6)
**Total**	**10 (0.2)**	**373 (8.1)**	**87 (1.9)**	**2341 (51)**	**1436 (31.3)**	**345 (7.5)**	**2724 (59.3)**	**1523 (33.2)**	**345 (7.5)**

Abbreviations: AD, Alzheimer's Disease; Aβ42, Amyloid β1–42; pTau, phosphorylated tau; Tau, total tau; APOE, genotype. 0 ε4 alleles, no ε4 (e.g., ε2/ε2, ε2/ε3, ε3/ε3); 1 ε4 allele, one ε4 (e.g., ε2/ε4, ε3/ε4); 2 ε4 alleles, two ε4 (e.g., ε4/ε4).

© 2025 Xu X et al. *JAMA Network Open*.

eTable 3. Distribution of CSF biomarkers by study cohort

Study	Aβ42, pg/mL, median (IQR)	Phosphorylated tau, pg/mL, median (IQR)	Total tau, pg/mL, median (IQR)
**ADNI**	158 (132-220)	34 (22-49)	75 (52-113)
**AIBL**	794 (607-954)	52 (42-68)	247 (185-329)
**Barcelona1**	633 (508-752)	97 (72-120)	705 (530-877)
**BIOCARD**	441 (382-464)	29 (24-43)	64 (47-74)
**Blennow**	502 (370-674)	78 (46-94)	595 (309-881)
**FACE**	618 (504-930)	70 (46-101)	461 (250-683)
**Homburg**	71 (63-86)	NA	77 (59-109)
**Knight ADRC**	618 (416-804)	48 (34-76)	264 (170-494)
**Lleo**	337 (251-490)	34 (26-45)	108 (67-135)
**London**	681 (485-952)	40 (29-62)	319 (224-491)
**MAYO**	336 (253-408)	18 (15-23)	81 (60-112)
**Molineuvo**	429 (341-580)	79 (55-107)	455 (266-732)
**NACC**	287 (179-447)	47 (29-88)	115 (54-563)
**Sweden**	265 (204-320)	94 (77-125)	724 (560-940)
**Upenn**	230 (184-253)	21 (15-28)	45 (38-80)
**UW**	162 (140-181)	42 (36-52)	41 (30-56)
**VMAP**	796 (566-938)	52 (43-67)	328 (263-472)
**WiscADRC**	684 (503-845)	43 (32-57)	297 (217-418)
**WRAP**	866 (620-1192)	17 (13-21)	195 (157-242)
**Zetter**	500 (409-965)	82 (64-107)	413 (334-636)
**Total**	**335 (182-638)**	**40 (25-64)**	**174 (75-397)**

Abbreviations: AD, Alzheimer's Disease; Aβ42, Amyloid β1–42; pTau, phosphorylated tau; Tau, total tau; SD, standard deviation; IQR, interquartile range. Median and interquartile range (IQR) for Aβ42, pTau, and Tau values, was calculated based on samples by cohorts.

© 2025 Xu X et al. *JAMA Network Open*.

eTable 4. Sex effect within and across ancestry

Trait	Model	European Ancestry		African Ancestry		Asian Ancestry		Meta-analysis across ancestries	
		**β (SE)**	**P Value**	**β (SE)**	**P Value**	**β (SE)**	**P Value**	**β (SE)**	**P Value**
Aβ42	Sex	-0.00 (0.03)	$9.24x10^{-1}$	0.27 (0.19)	$1.44x10^{-1}$	-0.28 (0.32)	$3.93x10^{-3}$	-0.58 (0.02)	$3.5x10^{-162}$
pTau	Sex	0.08 (0.03)	$7.25x10^{-3}$	0.45 (0.17)	$1.01x10^{-2}$	0.60 (0.32)	$4.31x10^{-1}$	0.35 (0.02)	$1.70x10^{51}$
Tau	Sex	0.12 (0.03)	$1.43x10^{-4}$	0.55 (0.21)	$9.28x10^{-3}$	0.60 (0.35)	$3.60x10^{-1}$	0.32 (0.02)	$3.10x10^{46}$

Abbreviations: Aβ42, Amyloid β1–42; pTau, phosphorylated tau; Tau, total tau. Sex, evaluates the main effect of the sex on CSF biomarkers from model 1.

© 2025 Xu X et al. *JAMA Network Open*.

eTable 5. Meta-analysis across ancestry

CSF	Variable	Fixed			Random			Heterogeneity	
		β	SE	P Value	β	SE	P Value	I^2 (%)	P Value
Aβ42	Sex	0	0.03	$9.40x10^{-01}$	0.03	0.1	$7.50x10^{-01}$	47.3	$1.50x10^{-01}$
Aβ42	APOE_ε4	-0.58	0.02	$3.50x10^{-162}$	-0.61	0.06	$1.10x10^{-22}$	0	$3.68x10^{-01}$
Aβ42	Sex × APOE_ε4	0.11	0.04	$1.40x10^{-02}$	0.11	0.04	$1.40x10^{-02}$	58.7	$8.87x10^{-02}$
Aβ42	APOE_ε4 in males	-0.63	0.03	$7.00x10^{-90}$	-0.63	0.03	$8.20x10^{-90}$	36.5	$2.07x10^{-01}$
Aβ42	APOE_ε4 in females	-0.53	0.03	$2.10x10^{-75}$	-0.6	0.09	$3.80x10^{-11}$	35.3	$2.13x10^{-01}$
PTAU	Sex	0.1	0.03	$7.10x10^{-04}$	0.25	0.14	$7.50x10^{-02}$	67.1	$4.81x10^{-02}$
PTAU	APOE_ε4	0.35	0.02	$1.70x10^{-51}$	0.35	0.02	$1.70x10^{-51}$	0	$5.90x10^{-01}$
PTAU	Sex × APOE_ε4	0.06	0.05	$2.30x10^{-01}$	0.34	0.26	$1.90x10^{-01}$	68.4	$4.23x10^{-02}$
PTAU	APOE_ε4 in males	0.32	0.03	$1.10x10^{-21}$	0.14	0.18	$4.60x10^{-01}$	65.6	$5.47x10^{-02}$
PTAU	APOE_ε4 in females	0.38	0.03	$3.30x10^{-31}$	0.43	0.1	$9.20x10^{-06}$	27.5	$2.52x10^{-01}$
TAU	Sex	0.12	0.03	$1.80x10^{-05}$	0.28	0.16	$8.00x10^{-02}$	63.4	$6.51x10^{-02}$
TAU	APOE_ε4	0.32	0.02	$3.10x10^{-46}$	0.32	0.02	$3.10x10^{-46}$	0	$6.67x10^{-01}$
TAU	Sex × APOE_ε4	0.1	0.05	$2.60x10^{-02}$	0.3	0.23	$1.90x10^{-01}$	51	$1.30x10^{-01}$
TAU	APOE_ε4 in males	0.27	0.03	$2.30x10^{-16}$	0.27	0.03	$2.40x10^{-16}$	54.4	$1.11x10^{-01}$
TAU	APOE_ε4 in females	0.37	0.03	$4.30x10^{-32}$	0.41	0.09	$2.20x10^{-06}$	0	$4.00x10^{-01}$

Abbreviations: Aβ42, Amyloid β1–42; pTau, phosphorylated tau; Tau, total tau; I^2, Inconsistency Index. Sex, evaluates the main effect of the sex on CSF biomarkers from model 1; APOE_ε4, evaluates the main effect of the APOE-ε4 allele on CSF biomarkers from model 1; Males, focuses on the effect of APOE-ε4 in males from model 2; Females, focuses on the effect of APOE-ε4 in females from model 3; Interaction, evaluates the interaction between sex and APOE-ε4 on CSF biomarkers from model 4. Fixed, fixed effect; random, random effect.

Random-effects model prioritized when significant heterogeneity across ancestries was observed ($P < 0.05$); otherwise, fixed-effect meta-analysis models were used.

© 2025 Xu X et al. *JAMA Network Open*.

eTable 6. Sensitivity analysis with ε3/ε3 as reference

		European Ancestry			African Ancestry			Asian Ancestry		
**Trait**	**Model**	**β**	**SE**	**P Value**	**β**	**SE**	**P Value**	**β**	**SE**	**P Value**
Aβ42	Sex	0	0.03	9.24E-01	0.27	0.19	1.44E-01	-0.28	0.32	3.93E-01
Aβ42	APOE_ε4	-0.55	0.02	2.64E-127	-0.76	0.14	8.93E-07	-0.67	0.24	7.55E-03
Aβ42	Males	-0.61	0.03	4.79E-72	-0.81	0.24	2.05E-03	0.43	0.63	5.07E-01
Aβ42	Females	-0.50	0.03	7.09E-58	-0.65	0.18	6.87E-04	-0.85	0.23	1.44E-03
Aβ42	Interaction	0.11	0.04	1.31E-02	0.18	0.30	5.61E-01	-1.33	0.63	4.19E-02
pTau	Sex	0.08	0.03	7.25E-03	0.45	0.17	1.01E-02	0.6	0.32	6.80E-02
pTau	APOE_ε4	0.33	0.02	1.17E-40	0.45	0.13	9.92E-04	0.19	0.24	4.30E-01
pTau	Males	0.31	0.04	1.78E-18	0.10	0.19	5.96E-01	-0.56	0.46	2.36E-01
pTau	Females	0.35	0.03	5.50E-24	0.66	0.18	5.45E-04	0.32	0.31	3.29E-01
pTau	Interaction	0.03	0.05	4.83E-01	0.55	0.27	4.68E-02	0.89	0.64	1.72E-01
Tau	Sex	0.12	0.03	1.43E-04	0.55	0.21	9.28E-03	0.6	0.35	9.17E-02
Tau	APOE_ε4	0.31	0.02	1.67E-37	0.47	0.16	4.38E-03	0.23	0.26	3.77E-01
Tau	Males	0.26	0.03	6.36E-14	0.21	0.24	3.87E-01	-0.71	0.44	1.25E-01
Tau	Females	0.35	0.03	9.46E-26	0.66	0.22	4.73E-03	0.42	0.35	2.52E-01
Tau	Interaction	0.09	0.05	5.97E-02	0.45	0.34	1.84E-01	1.08	0.69	1.26E-01

Abbreviations: Aβ42, Amyloid β1–42; pTau, phosphorylated tau; Tau, total tau. Sex, evaluates the main effect of the sex on CSF biomarkers from model 1; APOE_ε4, evaluates the main effect of the APOE-ε4 allele on CSF biomarkers from model 1; Males, focuses on the effect of APOE-ε4 in males from model 2; Females, focuses on the effect of APOE-ε4 in females from model 3; Interaction, evaluates the interaction between sex and APOE-ε4 on CSF biomarkers from model 4.

First sensitivity analyses were conducted by comparing APOE-ε4 carriers to APOE-ε3/ε3 homozygotes. In this analysis, APOE-ε4 was coded as follows: 0 for 3/ε3 , 1 for ε3/ε4 and ε2/ε4 , and 2 for ε4/ε4.

© 2025 Xu X et al. *JAMA Network Open*.

eTable 7. Sensitivity analysis without any APOE-ε2 carriers

		European Ancestry			African Ancestry			Asian Ancestry		
**Trait**	**Model**	**β**	**SE**	**P Value**	**β**	**SE**	**P Value**	**β**	**SE**	**P Value**
Aβ42	Sex	0	0.03	9.75E-01	0.29	0.19	1.29E-01	-0.28	0.32	3.93E-01
Aβ42	APOE_ε4	-0.55	0.02	4.32E-127	-0.74	0.15	1.88E-06	-0.67	0.24	7.55E-03
Aβ42	Males	-0.61	0.03	1.17E-71	-0.81	0.24	2.05E-03	0.43	0.63	5.07E-01
Aβ42	Females	-0.50	0.03	5.18E-58	-0.63	0.19	1.27E-03	-0.85	0.23	1.44E-03
Aβ42	Interaction	0.11	0.04	1.49E-02	0.20	0.30	5.17E-01	-1.33	0.63	4.19E-02
pTau	Sex	0.08	0.03	7.50E-03	0.44	0.17	1.32E-02	0.6	0.32	6.80E-02
pTau	APOE_ε4	0.34	0.02	6.71E-42	0.44	0.14	1.53E-03	0.19	0.24	4.30E-01
pTau	Males	0.32	0.04	5.50E-19	0.10	0.19	5.96E-01	-0.56	0.46	2.36E-01
pTau	Females	0.36	0.03	9.37E-25	0.64	0.18	9.05E-04	0.32	0.31	3.29E-01
pTau	Interaction	0.04	0.05	4.72E-01	0.54	0.28	5.49E-02	0.89	0.64	1.72E-01
Tau	Sex	0.12	0.03	1.54E-04	0.57	0.21	7.58E-03	0.6	0.35	9.17E-02
Tau	APOE_ε4	0.31	0.02	8.14E-39	0.49	0.16	3.44E-03	0.23	0.26	3.77E-01
Tau	Males	0.26	0.03	2.16E-14	0.21	0.24	3.87E-01	-0.71	0.44	1.25E-01
Tau	Females	0.36	0.03	1.21E-26	0.68	0.22	3.48E-03	0.42	0.35	2.52E-01
Tau	Interaction	0.09	0.05	5.75E-02	0.47	0.33	1.60E-01	1.08	0.69	1.26E-01

Abbreviations: Aβ42, Amyloid β1–42; pTau, phosphorylated tau; Tau, total tau. Sex, evaluates the main effect of the sex on CSF biomarkers from model 1; APOE_ε4, evaluates the main effect of the APOE-ε4 allele on CSF biomarkers from model 1; Males, focuses on the effect of APOE-ε4 in males from model 2; Females, focuses on the effect of APOE-ε4 in females from model 3; Interaction, evaluates the interaction between sex and APOE-ε4 on CSF biomarkers from model 4.

Second sensitivity analyses were conducted by comparing APOE-ε4 carriers to APOE-ε3/ε3 homozygotes and removing APOE-ε2/4 heterozygotes. Specifically, in this analysis, APOE-ε4 was coded as follows: 0 for ε3/ε3; 1 for ε3/ε4; and 2 for ε4/ε4.

© 2025 Xu X et al. *JAMA Network Open*.

eFigure 1. Flow chart

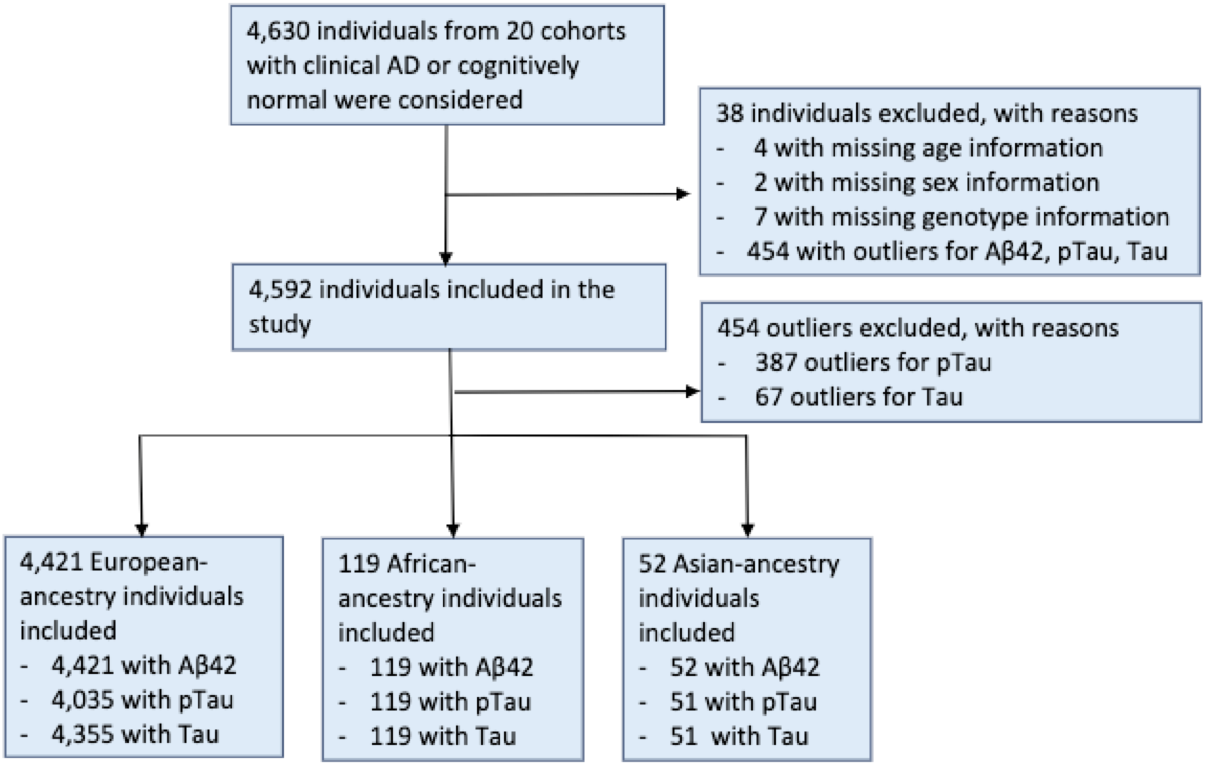

Abbreviations: Aβ42, Amyloid β1–42; pTau, phosphorylated tau; Tau, total tau.

This flowchart illustrates the inclusion process for participants from 20 cohorts. Out of 4,630 individuals, our analysis included 4,421 individuals of European ancestry, 119 individuals of African ancestry, and 52 individuals of Asian ancestry. This classification ensures the robust analysis of CSF biomarkers across groups.

© 2025 Xu X et al. *JAMA Network Open*.

eFigure 2. Genetically derived ancestry information

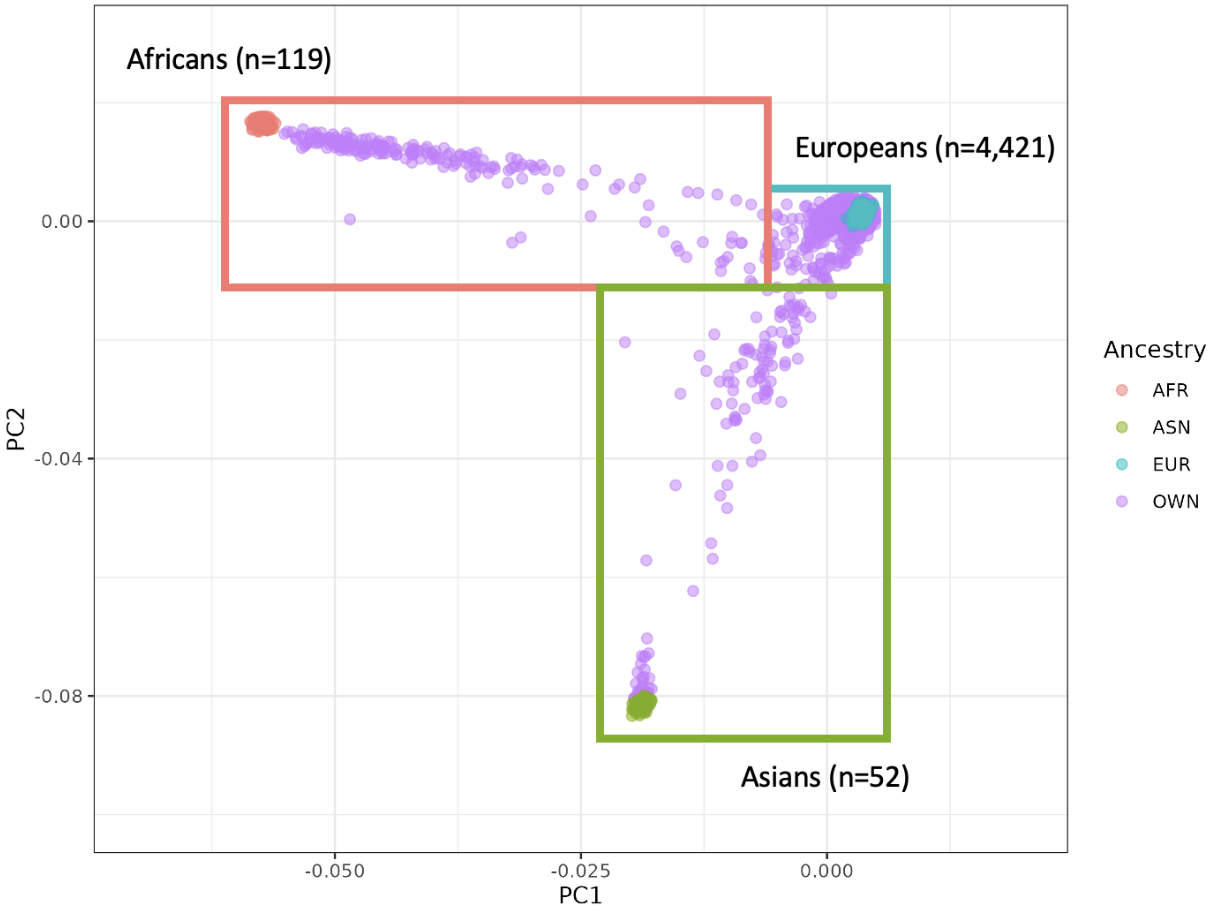

This figure presents the genetically derived information by ancestry. To determine ancestry, we used the first two principal components (PC) from the principal component analysis (PCA). By anchoring HapMap data with European (EUR; in cyan), African (AFR, in orange) and Asian (ASN, in green), samples across 20 cohorts (OWN, in purple) were grouped to European (n=4,421, in cyan box). African (n=119, in orange box) and Asian (n=52, in green box).

© 2025 Xu X et al. *JAMA Network Open*.

eFigure 3. Histogram of CSF biomarkers before and after harmonization

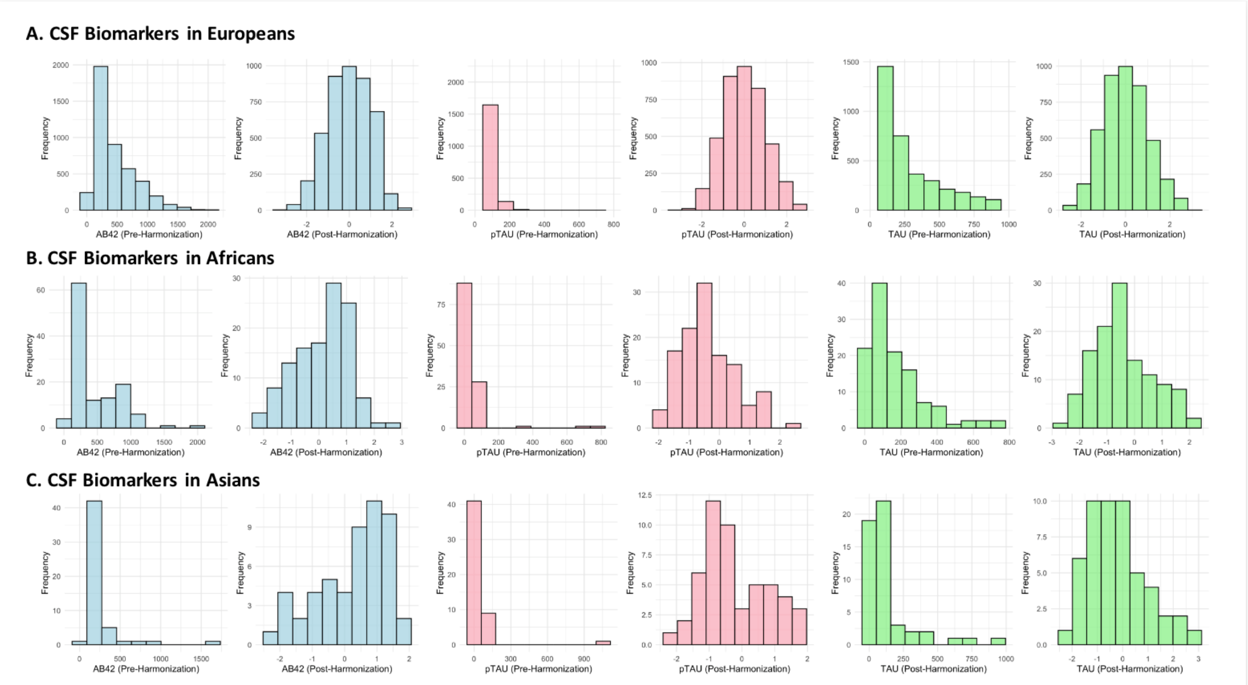

This figure presents the distribution of cerebrospinal fluid (CSF) biomarkers (Aβ42, pTau, and Tau) before and after harmonization, stratified by ancestry groups: Europeans (Panel A), Africans (Panel B), and Asians (Panel C). Pre-harmonization data displays a skewed distribution. Post-harmonization, applying log10 transformation and Z-score normalization, improves distributions. This highlights ancestry-specific variations and the effect of harmonization.

© 2025 Xu X et al. *JAMA Network Open*.

eFigure 4. Sex and APOE-ε4 effect across both sexes

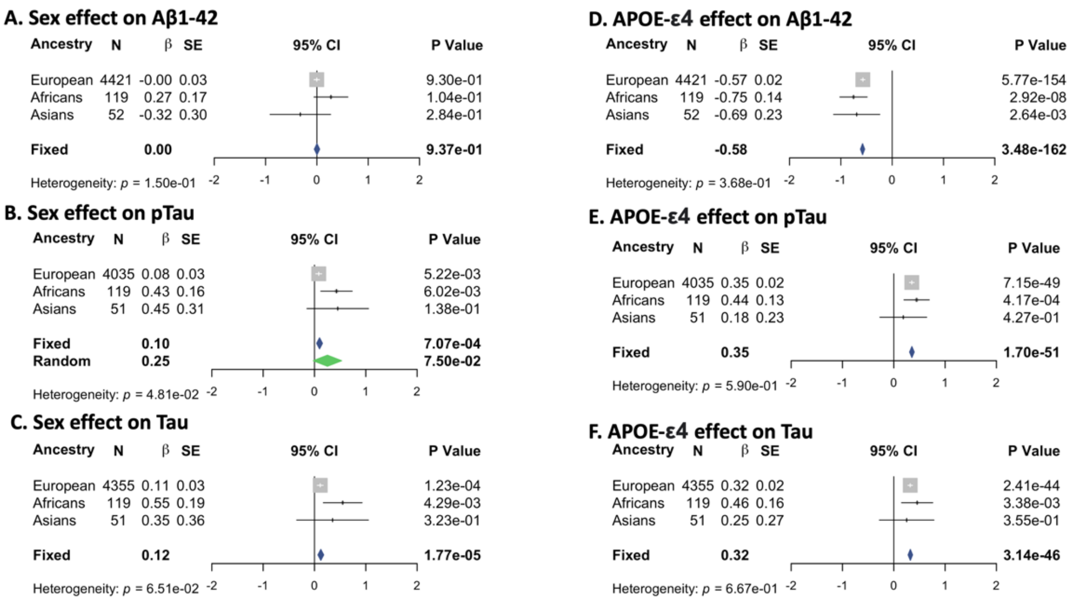

For CSF biomarkers Aβ1-42, pTau, and Tau, the main effect of sex (Panel A, B, C) and APOE-ε4 (Panel D, E, F) were analyzed using the model $Y = \alpha + \beta_1 \times APOE\_\varepsilon4 + \beta_2 \times Sex + \beta_3 \times Age + \varepsilon$ were presented. The results are stratified by ancestry, including European, African, and Asian populations, and are displayed in order of effect size. Each effect is represented by a square proportional to the sample size, along with its 95% confidence interval indicated by horizontal lines. Fixed-effect meta-analyses were performed for all three biomarkers, as heterogeneity tests across ancestries were not significant (P > .05).
However, for pTau, heterogeneity was significant (P = 4.81×10^{-2}), necessitating both fixed-effect (blue diamond) and random-effect (green diamond) meta-analyses.

© 2025 Xu X et al. *JAMA Network Open*.

eFigure 5. Sex-stratified APOE-ε4 effects

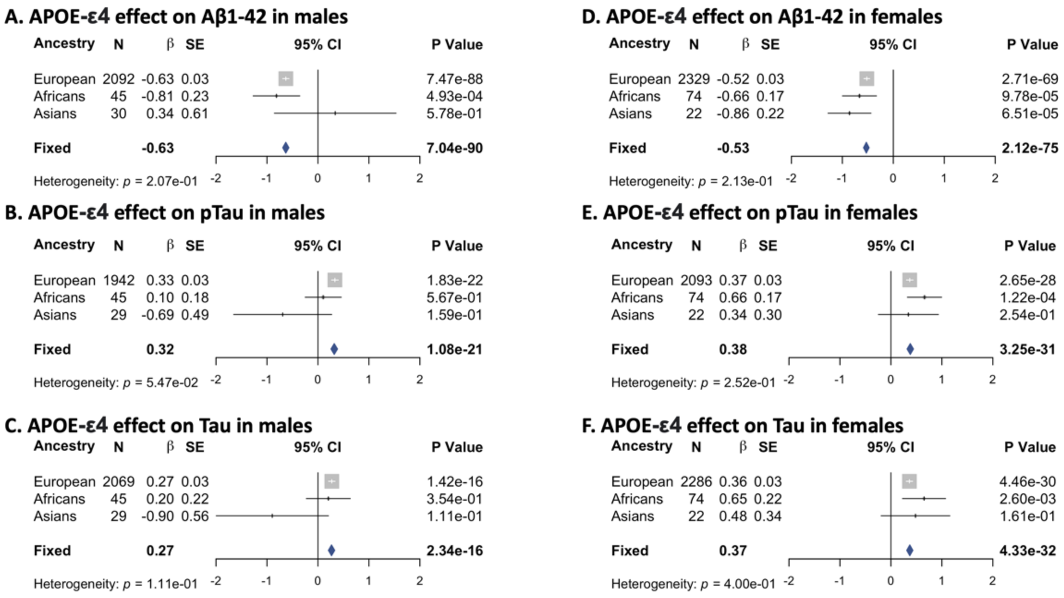

For CSF biomarkers Aβ1-42, pTau, and Tau, the effect of APOE-ε4 was analyzed separately in males using the stratified models $Y = a + \beta_1 \times APOE\_\varepsilon 4 + \beta_2 \times Age + \varepsilon$. The results, stratified by ancestry (European, African, and Asian), are presented in Panels A-F, with effects displayed in order of effect size. Each effect is represented by a square proportional to the sample size, with horizontal lines indicating the 95% confidence intervals.

Fixed-effect meta-analyses (blue diamond) were conducted across ancestries. This stratified analysis highlights potential differences in APOE-ε4 effects on CSF biomarkers between sexes across different ancestry groups.

© 2025 Xu X et al. *JAMA Network Open*.

eFigure 6. Interaction effect between sex and APOE-ε4

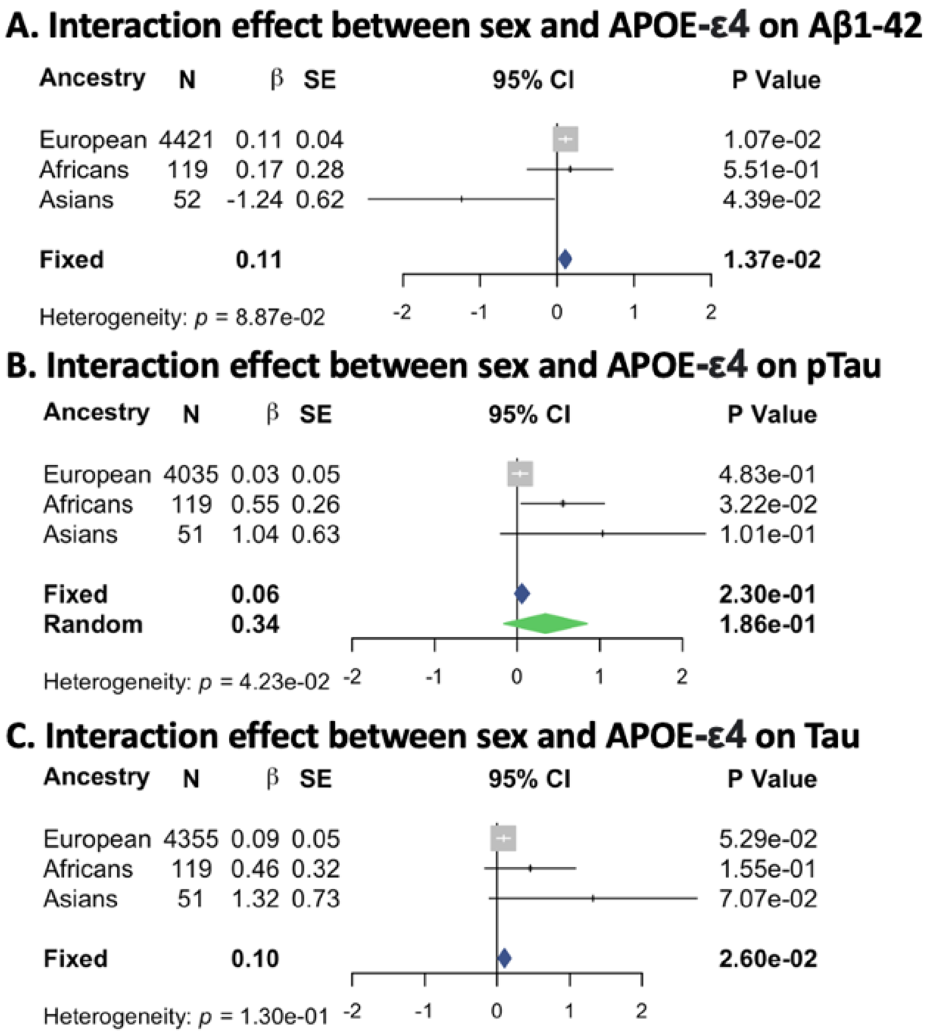

For the CSF biomarkers Aβ1-42, pTau, and Tau, the interaction effects between sex and APOE-ε4 across ancestries were accessed using the model $Y = a + \beta_1 \times APOE\_\varepsilon4 + \beta_2 \times Sex + \beta_3 \times Age + \beta_4 \times APOE\_\varepsilon4 \times Sex + \varepsilon$. The results, stratified by ancestry (European, African, and Asian), are presented in Panels A-C, with interaction effects displayed in order of effect size. Each interaction effect is represented by a square proportional to the sample size, with horizontal lines indicating the 95% confidence intervals.

For Aβ1-42 (Panel A) and Tau (Panel C) , fixed-effect meta-analyses were performed due to the absence of significant heterogeneity across ancestries (P>.05), as indicated by the blue diamond.

For pTau (Panel B), heterogeneity across ancestries was significant (P=4.23×10−2), necessitating both fixed-effect (blue diamond) and random-effect (green diamond) meta-analyses.

© 2025 Xu X et al. *JAMA Network Open*.

eFigure 7. Sex-stratified APOE-ε4 effect in Asian ancestry

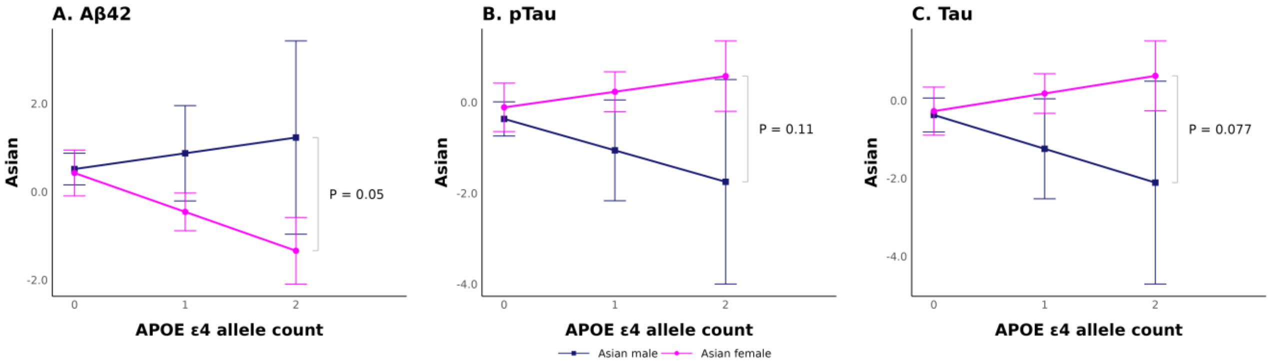

For CSF biomarkers Aβ42, pTau, and Tau, the interaction effect of sex and APOE-ε4 (Panels A, B, and C, respectively) in individuals of Asian ancestry was analyzed using stratified models. Specifically, Model 2 ($Y = a + \beta_1 \times \mathrm{APOE}_{\varepsilon 4} + \beta_2 \times Age + \varepsilon$ (stratified analysis with males only)) was applied to males, and Model 3 ($Y = a + \beta_1 \times \mathrm{APOE}_{\varepsilon 4} + \beta_2 \times Age + \varepsilon$ (stratified analysis with females only)) was applied to females. The predicted biomarker levels, stratified by sex, are shown as separate lines for males (black) and females (pink), with error bars representing 95% confidence intervals.

The x-axis represents the APOE-ε4 allele count (0, 1, or 2), while the y-axis shows the predicted normalized biomarker levels. The plots display separate trajectories for males (black) and females (pink), with error bars representing 95% confidence intervals.

© 2025 Xu X et al. *JAMA Network Open*.

eMethods 1. Cohort description

Alzheimer's Disease Neuroimaging Initiative (ADNI)

Data used in the analyses performed in this article were obtained from the Alzheimer's Disease Neuroimaging Initiative (ADNI) database (adni.loni.usc.edu). ADNI was launched in 2003 as a public-private partnership, led by Principal Investigator Michael W. Weiner, MD. The primary goal of ADNI has been to test whether serial magnetic resonance imaging (MRI), positron emission tomography (PET), other biological markers, and clinical and neuropsychological assessment can be combined to measure the progression of mild cognitive impairment (MCI) and early Alzheimer's disease (AD). For up-to-date information, see www.adni-info.org.

Australian Imaging, Biomarkers and Lifestyle (AIBL)

Australian Imaging, Biomarker & Lifestyle Flagship Study of Ageing (AIBL) study was initiated in November 2006 with a prospective longitudinal study design, intending to assess all participants at 18-month intervals. Over 1100 participants, comprising of healthy controls (HC), individuals with mild cognitive impairment (MCI) and Alzheimer's disease (AD) were evaluated over 4.5 years. Data collection was done in two centers with 40% of subjects from Perth in Western Australia and 60% from Melbourne, Victoria. Neuroimaging was planned for 25% of each of these groups. However, the actual enrollment in the neuroimaging arm included 177 HC, 57 MCI, and 53 individuals with mild AD, constituting 26% of the entire cohort[1]. All individuals in the study were aged 60 or older, and they were in generally good health, with no prior history of stroke or any other neurological conditions. Those diagnosed with AD met the criteria outlined by the National Institute of Neurological and Communicative Disorders–Alzheimer's Disease and Related Disorders (NINCDS-ADRDA) for probable AD, as defined by McKhann et al in 19844. Additionally, these AD patients exhibited a Clinical Dementia Rating (CDR) of 1 or higher. For additional information see https://aibl.csiro.au/.

Barcelona-1

Barcelona -1 is a longitudinal observational study consisting of ~300 subjects at baseline carried out in the Memory and Disorder unit at the University Hospital Mutua de Terrassa, Terrassa, Barcelona, Spain. Cases include subjects diagnosed with AD dementia (ADD), non-AD dementias (non-ADD), mild cognitive impairment (MCI), or subjective memory complaints (SMC). Clinical information was collected at baseline as well as longitudinally and lumbar puncture (LP) and amyloid PET were performed if subjects had diagnosis of MCI, early-onset dementia (<65 years), or dementia with atypical clinical features[3].

Biomarkers for Older Controls at Risk for Dementia (BIOCARD)

The Biomarkers for Older Controls at Risk for Dementia (BIOCARD) study is a longitudinal study that was initiated in 1995 in the National Institute of Mental Health (NIMH) Intramural Research Program. The study encompassed clinical, neuropsychological, and neuropsychiatric evaluations, neuroimaging, and fluid biomarkers assessment to understand and predict progression from normal cognition to mild cognitive impairment (MCI) and dementia, particularly Alzheimer's disease (AD). The average age at enrollment was 55 years. The study was stopped in 2005, however, in 2009 it was re-started at Johns Hopkins University (JHU). Between 1995 and 2005, clinical and cognitive assessments were performed annually, and CSF, blood sampling, and magnetic resonance imaging (MRI) were done every 2 years[2]. From 2009 until

© 2025 Xu X et al. *JAMA Network Open*.

2015, participants were annually evaluated for clinical, and cognitive assessments and blood sample collection with bi-annual MRI and CSF being restarted since 2015[2].

Ace Alzheimer Center Barcelona (FACE)

We obtained cerebrospinal biomarker data from Ace Alzheimer Center Barcelona also known as Fundació ACE (FACE) which is a leading non-profit research and clinical center specializing in Alzheimer's disease and other neurodegenerative disorders. Founded in 1995 and headquartered in Barcelona, this center combines both research activities and clinical care services. To date, the center has diagnosed over 30,000 patients, collected 20,000 blood and 1,831 cerebrospinal fluid samples, analyzed almost 13,000 genetic samples, and participated in almost 150 clinical trials during its existence[4]. For more details, visit http://www.fundacioace.com/en.

Saarland University in Homburg/Saar, Germany (Homburg)

CSF biomarker data from 107 subjects were collected at Saarland University in Germany. These included demented outpatients who referred to a hospital memory clinic between 1995 and 2001 for diagnostic evaluation [5]. For clinical diagnosis of probable AD, the National Institute of Neurological and Communication Disorders and Stroke/AD and Related Disorders Association criteria were applied [5]. Additional evaluation performed included CSF and blood sample collection, assessment of cognitive impairment, APOE genotyping among others. Age at onset was defined by the appearance of the first clinical symptoms [5].

Knight ADRC

Charles F. and Joanne Knight Alzheimer's Disease Research Center (Knight ADRC), housed at Washington University in St. Louis, is one of 30 ADRCs funded by NIH. The goal of this collaborative research effort is to advance AD research with the goal of treatment or prevention of AD. The subjects included in this study are from the Memory and Aging Project (MAP) supported by Knight ADRC. As part of the project, subjects undergo annual psychometric testing and interviews along with biennial or triennial PET, MRI, and CSF collection. Further details on Knight ADRC and MAP can be found at https://knightadrc.wustl.edu/.

Hospital Sant Pau (Lleo)

The memory unit of Hospital Sant Pau located in Barcelona, Spain, attends more than 2000 patients annually. Routine assessment includes neuropsychological evaluation, CSF blood sample collection and MRI tests. Additionally, PET examination is conducted in a select group of subjects. The subjects included in this study were recruited from the year 2009 to 2016[6]. Additional information on the study can be found at https://santpaumemoryunit.com/.

London

The CSF biomarker data under the London cohort were from EDAR and DESCRIPA studies. EDAR is a prospective, longitudinal study aimed at examining and evaluating biomarkers of early AD[8,9]. In particular, the study is focused on Aβ oligomers and the effect of genetic variants on these oligomers. More information on the cohort can be found at http://www.edarstudy.eu. DESCRIPA study is also a prospective, multicenter study. Led by the European AD Consortium, the focus of the study is on collecting data from non-demented subjects to develop screening

and diagnostic criteria for AD[9]. Further details of this study can be found in Visser et. al, (2008)[10].

MAYO

The CSF biomarker data under the Mayo cohort were collected by biospecimen accessioning and processing core at Mayo Clinic in Rochester, Minnesota. The research core at Mayo Clinic usually serves study groups affiliated with the clinic, however, some facilities are available to investigators worldwide. The Proteomics Core in particular offers routine services to identify and quantify proteins. Longitudinal CSF samples were available from 443 individuals comprising close to 100% control group.

Clinic de Barcelona (Molineuvo).

CSF samples in this cohort were collected from individuals recruited at the Alzheimer's disease and other cognitive disorder unit, from the Hospital Clinic de Barcelona (Barcelona, Spain)[12]. CSF samples were collected from 256 individuals with 36.8% being male. In addition to CSF profiling, an assessment of cognitive impairment was also performed as part of data collection. Samples were provided by Dr. Molineuvo.

National Alzheimer's Coordinating Center (NACC)

The National Alzheimer's Coordinating Center (NACC) is the central data repository for all NIA's Alzheimer's Disease and Research Centers (ADRC) Programs, including Mayo ADRC and Knight ADRC. The CSF biomarker data utilized from NACC in this paper were selected after proper IBD to make sure no duplicates or close relatives of samples from other ADRC cohorts were included in GWAS. Currently, NACC houses data from 47000 participants from across 33 ADRCs and 4 exploratory centers. The data is collected using a prospective, standardized, and longitudinal clinical evaluation of subjects. NACC protocol requires annual follow-up for as long as the participant can be involved and is thus longitudinal. CSF as well as Imaging data are available as a part of the data repository. However, the data collection schedule and subject enrollment protocol vary by center. For additional information please check this link https://naccdata.org/.

Skåne University Hospital, Sweden (SWEDEN)

The samples included in this cohort were evaluated at the memory disorder unit at Skåne University Hospital. Of the total 315 subjects included in this study, 100% of them were diagnosed as having AD. Details of sample collection in this cohort have been described previously[13]. Briefly, at the thorough physical, neurological, and psychiatric examination, as well as a clinical interview focusing on cognitive symptoms are performed as baseline visit. Furthermore, cognitive tests, analysis of APOE genotype, and imaging of the brain were also done. Additional information on the center can be found at https://vard.skane.se/en/skane-university-hospital/.

University of Pennsylvania (UPENN)

The CSF biomarker data used in this study were received from the University of Pennsylvania's Alzheimer's Disease Research Center. Participants should be of age 55 years or older to be enrolled in the cohort. Annual visit includes cognitive testing, neurological exam, blood samples and interviews. MRI and PET scan data are available for a subset of the samples. The

© 2025 Xu X et al. *JAMA Network Open*.

longitudinal biomarker data used in this study were from 182 unique participants and were measured using the Luminex platform.

University of Washington (UW)

The UW Alzheimer's Disease Research Center, located in Seattle, is part of one of the NIH-funded ADRCs focused on Alzheimer's disease and related dementia. The center conducts yearly visits during which participants are required to complete tests of their memory and thinking and have a brief physical and neurological examination. Blood draw is usually part of the annual visits, but CSF collection is voluntary. For additional information on the center please visit https://depts.washington.edu/mbwc/adrc.

Vanderbilt Memory and Aging Project (VMAP)

Founded in 2012, the Vanderbilt Memory and Aging Project (VMAP) is a longitudinal study focused on the study of brain aging. As part of the project, physical and frailty examination, fasting blood draw, neuropsychological assessment, echocardiogram, cardiac MRI, CSF collection and brain MRI are usually obtained at baseline visits. Follow-up visits are then scheduled at 18 months, 3 years, 5 years, and so on. To be a part of the study, individuals must be 50+ years old and show no signs of cognitive decline. More details on the project can be found at https://www.vumc.org/vmac/home.

Wisconsin Alzheimer's Disease Research Center (WiscADRC)

The Wisconsin Alzheimer's Disease Research Center housed at the University of Wisconsin-Madison is one of the several NIH-funded ADRC centers in the USA. Established in 2009, the primary focus of the center is to improve early detection of Alzheimer's disease and find ways to delay onset and progression. As of 2023, the center has collected data from 1075 core participants of which 601 have undergone CSF collection and 572 have PET scans available. Cognitive assessment is done either annually or at every other year visit. Further details can be found at https://www.adrc.wisc.edu/.

Wisconsin Registry for Alzheimer's Prevention (WRAP)

The Wisconsin Registry for Alzheimer's Prevention is a longitudinal observational cohort study enriched with persons with a parental history (PH) of probable Alzheimer's disease (AD) dementia. Since late 2001, the Wisconsin Registry for Alzheimer's Prevention has enrolled 1561 people at a mean baseline age of 54 years. Participants return for a second visit 4 years after baseline, and subsequent visits occur every 2 years.

Note: Samples included in Blennow and Zetter cohorts were provided by Dr. Blennow and Dr. Zetterberg respectively. DNA samples along with CSF biomarker measurement, disease status, gender and age at sample collection information were provided. Additional information about these cohorts is not available current.

© 2025 Xu X et al. *JAMA Network Open*.

References:

1. Rowe CC, Ellis KA, Rimajova M, et al. Amyloid imaging results from the Australian Imaging, Biomarkers and Lifestyle (AIBL) study of aging. *Neurobiol Aging*. 2010;31(8):1275-1283. doi:10.1016/J.NEUROBIOLAGING.2010.04.007
2. Koychev I, Vaci N, Bilgel M, et al. Prediction of rapid amyloid and phosphorylated-Tau accumulation in cognitively healthy individuals. *Alzheimer's & Dementia : Diagnosis, Assessment & Disease Monitoring*. 2020;12(1). doi:10.1002/DAD2.12019
3. Álvarez I, Diez-Fairen M, Aguilar M, et al. Added value of cerebrospinal fluid multimarker analysis in diagnosis and progression of dementia. *Eur J Neurol*. 2021;28(4):1142-1152. doi:10.1111/ENE.14658
4. de Rojas I, Moreno-Grau S, Tesi N, et al. Common variants in Alzheimer's disease and risk stratification by polygenic risk scores. *Nature Communications 2021 12:1*. 2021;12(1):1-16. doi:10.1038/s41467-021-22491-8
5. Riemenschneider M, Wagenpfeil S, Diehl J, et al. Tau and Abeta42 protein in CSF of patients with frontotemporal degeneration. *Neurology*. 2002;58(11):1622-1628. doi:10.1212/WNL.58.11.1622
6. Sala I, Illán-Gala I, Alcolea D, et al. Diagnostic and Prognostic Value of the Combination of Two Measures of Verbal Memory in Mild Cognitive Impairment due to Alzheimer's Disease. *J Alzheimers Dis*. 2017;58(3):909-918. doi:10.3233/JAD-170073
7. Dagley A, LaPoint M, Huijbers W, et al. Harvard Aging Brain Study: Dataset and accessibility. *Neuroimage*. 2017;144(Pt B):255-258. doi:10.1016/J.NEUROIMAGE.2015.03.069
8. Macdonald SWS, Thorvaldsson V, Johansson B, et al. O3-02-03: Cognitive function and cognitive change in dementia, mild cognitive impairment, and healthy aging: The EDAR study. *Alzheimer's & Dementia*. 2010;6(4S_Part_4):S127-S127. doi:10.1016/J.JALZ.2010.05.399
9. Voyle N, Patel H, Folarin A, et al. Genetic Risk as a Marker of Amyloid-β and Tau Burden in Cerebrospinal Fluid. *Journal of Alzheimer's Disease*. 2017;55(4):1417. doi:10.3233/JAD-160707
10. Visser PJ, Verhey FRJ, Boada M, et al. Development of Screening Guidelines and Clinical Criteria for Predementia Alzheimer's Disease: The DESCRIPA Study. *Neuroepidemiology*. 2008;30(4):254. doi:10.1159/000135644
11. Campbell MC, Myers PS, Weigand AJ, et al. Parkinson disease clinical subtypes: key features & clinical milestones. *Ann Clin Transl Neurol*. 2020;7(8):1272-1283. doi:10.1002/ACN3.51102
12. Falcon C, Monté-Rubio GC, Grau-Rivera O, et al. CSF glial biomarkers YKL40 and sTREM2 are associated with longitudinal volume and diffusivity changes in cognitively unimpaired individuals. Published online 2019. doi:10.1016/j.nicl.2019.101801
13. Hertze J, Minthon L, Zetterberg H, Vanmechelen E, Blennow K, Hansson O. Evaluation of CSF biomarkers as predictors of Alzheimer's disease: a clinical follow-up study of 4.7 years. *J Alzheimers Dis*. 2010;21(4):1119-1128. doi:10.3233/JAD-2010-100207
14. Mathis CA, Kuller LH, Klunk WE, et al. In vivo assessment of amyloid-β deposition in nondemented very elderly subjects. *Ann Neurol*. 2013;73(6):751-761. doi:10.1002/ANA.23797

© 2025 Xu X et al. *JAMA Network Open*.
