# Supplementary material for: Sex Differences in Apolipoprotein E and Alzheimer Disease Pathology Across Ancestries
Source: JAMA Netw Open. 2025 Mar 11;8(3):e250562. doi: 10.1001/jamanetworkopen.2025.0562 (PMC11897841; doi:10.1001/jamanetworkopen.2025.0562)
Supplement: Supplement 2. — Data Sharing Statement [file jamanetwopen-e250562-s002.pdf]

Data Sharing Statement

Xu. Sex Differences in Apolipoprotein E and Alzheimer Disease Pathology Across Ancestries. *JAMA Netw Open*. Published March 11, 2025. doi:10.1001/jamanetworkopen.2025.0562

Data
Data available: No
